# MicroRNA-27b Regulates Mitochondria Biogenesis in Myocytes

**DOI:** 10.1371/journal.pone.0148532

**Published:** 2016-02-05

**Authors:** Linyuan Shen, Lei Chen, Shunhua Zhang, Jingjing Du, Lin Bai, Yi Zhang, Yanzhi Jiang, Xuewei Li, Jinyong Wang, Li Zhu

**Affiliations:** 1 College of Animal Science and Technology, Sichuan Agricultural University, Chengdu, 611130, Sichuan, China; 2 Chongqing Academy of Animal Science, Rongchang, 402460, Chongqing, China; 3 Department of Animal Science, Xichang College, Xichang, 615000, Sichuan, China; 4 Department of Biology, College of Life and Science, Sichuan Agricultural University, Chengdu, 611130, Sichuan, China; Tohoku University, JAPAN

## Abstract

MicroRNAs (miRNAs) are small, non-coding RNAs that affect the post-transcriptional regulation of various biological pathways. To date, it is not fully understood how miRNAs regulate mitochondrial biogenesis. This study aimed at the identification of the role of miRNA-27b in mitochondria biogenesis. The mitochondria content in C2C12 cells was significantly increased during myogenic differentiation and accompanied by a marked decrease of miRNA-27b expression. Furthermore, the expression of the predicted target gene of miRNA-27b, forkhead box j3 (*Foxj3*), was also increased during myogenic differentiation. Luciferase activity assays confirmed that miRNA-27b directly targets the 3’-untranslated region (3’-UTR) of *Foxj3*. Overexpression of miRNA-27b provoked a decrease of mitochondria content and diminished expression of related mitochondrial genes and *Foxj3* both at mRNA and protein levels. The expression levels of downstream genes of *Foxj3*, such as *Mef2c*, *PGC1α*, *NRF1* and *mtTFA*, were also decreased in C2C12 cells upon overexpression of miRNA-27b. These results suggested that miRNA-27b may affect mitochondria biogenesis by down-regulation of *Foxj3* during myocyte differentiation.

## Introduction

Mitochondria are essential eukaryotic organelles whose most important function is to provide the cellular chemical energy in form of ATP [1]. In addition, mitochondria also play important roles in other biological processes, such as amino acid metabolism and ion homeostasis [2]. However, mass, function, and morphology of mitochondria varies widely in different tissues and is dynamically regulated depending on nutrient availability and energy demand [3,4]. Skeletal muscle, for instance, comprises about 40% of our body mass and consumes more oxygen than liver, kidney and brain [5,6]. Any mitochondrial dysfunction may furthermore result in serious metabolic problems, as is the case in amyotrophic lateral sclerosis [7]. Reduced mitochondrial content in skeletal muscle is a pathogenic factor for type 2 diabetes [8]. Mitochondrial biogenesis in skeletal muscle is tightly regulated by the interaction of transcription factors such as the peroxisome proliferator-activated receptor gamma coactivator 1-alpha (PGC1-α), the nuclear respiratory factor 1 (NRF-1), forkhead box j3 (Foxj3), the myocyte enhancing factor-2C (Mef2c) and the mitochondrial transcription factor A (mtTFA) [9–12].

MicroRNA (miRNAs) are small, noncoding RNAs, usually 21–23 nucleotides in length, that negatively regulate protein expression by binding to the 3’ untranslated region (3’-UTR) of their target mRNA [13,14]. miRNAs have been observed to participate in the regulation of numerous biological processes, such as mitochondrial biogenesis in muscle tissue [15]. A further understanding of the control of mitochondrial biogenesis by miRNAs may not only close knowledge gaps regarding mitochondrial function but may also reveal potential therapeutic targets in mitochondria dysfunction diseases. In previous studies, miRNAs have been reported to regulate mitochondrial biogenesis in muscle tissue. miRNA-484, for instance, has been shown to suppress the translation of mitochondrial fission protein and thereby reduce mitochondrial fission, apoptosis and myocardial infarction [16]. Yamamoto *et al*. found that miRNA-494 may reduce mitochondrial biogenesis by down-regulating mtTFA and Foxj3 during myocyte differentiation [10]. In addition, Kang *et al*. demonstrated that miRNA-27b may impair adipocyte differentiation and mitochondria function by targeting prohibitin in human adipose-derived stem cells [17]. miRNA-27b has also been shown to play an important role in cardiac hypertrophy and fibrosis, pathological conditions characterized by mitochondria injury [18]. These previous results imply that miRNA-27b may have a potential functions in regulating mitochondrial biogenesis in muscle or myocytes. Furthermore, in an earlier study, we found miRNA-27b to be among the ten most highly expressed miRNAs in different muscle tissues and have a potential target gene of Foxj3 [19]. However, Foxj3 is the direct transcription factor upregulates the expression of Mef2c [20]. Mef2c plays important role in regulating muscle development and maintenance of mitochondria function [21–22].

In this study, a dual-luciferase reporter assay was used to investigate the direct target gene (*Foxj3*) of miRNA-27b and explore it’s function in mitochondrial biogenesis. In addition, we used synthetic miRNA mimics and inhibitors in gain- and loss-of-function experiments to investigate the roles of miRNA-27b in mitochondrial biogenesis. We provide evidence that miRNA-27b is involved in the regulation of mitochondrial biogenesis.

## Materials and Methods

### Cell culture

Mouse C2C12 myoblasts (CRL-1772) and human Hep-2 HeLa cell derivatives (ATCC CCL-23) were obtained from the American Type Culture Collection (ATCC, Rockville, MD, USA). Growth medium (GM) for C2C12 myoblasts was high glucose Dulbecco’s modified Eagle medium (DMEM) supplemented with 10% (v/v) fetal bovine serum (FBS). C2C12 cell culture in GM maintained cell’s in their undifferentiated state and able to proliferate. Upon reaching 80% of confluence, differentiation was induced by replacing GM with differentiation medium (DM) that consisted of DMEM supplemented with 2% horse serum. DM was replaced every 48 h. The human cervical carcinoma HeLa cells were maintained in DMEM supplemented with 10% FBS. Cell culture procedures were conducted in a humidified atmosphere at 37°C and 5% CO_2_.

### Cell transfection

C2C12 cells were seeded in 24-well plates and transfected with an miRNA-27b mimic, an miRNA-27b inhibitor or non-specific control oligonucleotides (Ribobio, Guangzhou, China), respectively, upon reaching 80% of confluence. Briefly, 2.5 μl LipofectamineTM2000 (Invitrogen, Carlsbad, CA, USA) were added to 50 μl serum-reduced medium (Opti-MEM) to yield solution A. For solution B, 2 μl miRNA-27b mimic (20 μM) or 3 μl miRNA-27b inhibitor (20 μM) were added to 50 μl Opti-MEM. Both solutions were incubated for 5 min at room temperature (RT) before being mixed both. After another 20 min of incubation, the mix was added to cells. After 6 h, the medium was changed. After 2 days of differentiation, the transfection was repeated. Cells were harvested after 5 days of differentiation.

### Real-time PCR

Total RNA (including miRNA) was extracted from C2C12 cells with TRIzol Reagent (Invitrogen, Carlsbad, CA, USA) according to the manufacturer’s instructions. In order to measure expression levels of mRNAs and miRNAs, reverse transcription was performed using PrimeScript RT Master Mix kit, and PrimeScript™ miRNA RT-PCR Kit (both obtained from TaKaRa, Dalian, China), respectively, following the manufacturer’s recommendations. Quantitative PCR was conducted by means of the SYBR Premix Ex Taq kit (TaKaRa) on the CFX96 system (Bio-Rad, Richmond, CA, USA). In brief, a Ploy(A) tail was added to the miRNA, which was then transcribed into cDNA using a universal adaptor primer that included oligo-dT. The generated cDNA was then combined with the Uni-miR qPCR Primer (possess the binding site with universal adaptor primer, included in kit) and a miRNA primer (sequence complementary to the miRNA) to complete the Real time PCR reaction. miRNA RT-PCR cycle parameters were as follows: 95°C for 30 seconds, followed by 44 cycles at 95°C for 5 seconds and 60°C for 35 seconds. Real time PCR for mRNAs was essentially conducted as reported before [23]. Relative expression levels of mRNAs and miRNAs were calculated using the 2^-ΔΔCt^ method [24]. Expression levels of U6 and β-actin were used as endogenous controls and for normalization of the expression of miRNA and mRNA, respectively. Total DNA was extracted from C2C12 cells using DNeasy (Qiagen, Hataworth, CA, USA) in accordance with the manufacturer’s instructions. The relative expression ratio of the mitochondrial ND2, ATP6 and COX I to the nuclear B2M was calculated in order to estimate total mtDNA content. The relative mtDNA copy number per diploid cell was estimated to 2^ΔCt^. Primer sequences are shown in Table 1.

**Table 1:** Primers used for PCR.

Gene Symbol	Gene ID	Primer Sequence (5'-3')
***Foxj3***	230700	F-AGCCTAACATCTATGGACTGGT
		R-GGTCAAGGAGTGCATTCTTCTTA
***MEF2c***	17260	F-ATCTCTCCCTGCCTTCTACTC
		R-CTCCCATCGTAGGAACTGCT
***PGC1-α***	19017	F-AAGGTCCCCAGGCAGTAGAT
		R-AAGGGAGAATTGCGGTGTGT
***NRF1***	18181	F-TCTCACCCTCCAAACCCAAC
		R-CCCGACCTGTGGAATACTTG
***mtTFA***	21780	F-CCCCTCGTCTATCAGTCTTGT
		R-CTGCTTCTGGTAGCTCCCTC
***ND2***	17717	F-CGCCCCATTCCACTTCTGATTACC
		R-TTAAGTCCTCCTCATGCCCCTATG
***ATP6***	17705	F-TGCTGGATAAGCTCGTGCTC
		R-GGTCAACCTGTACCTCCAAATG
***COX I***	17708	F-TCACGTCTGTCACTGCCATTA
		R-GGGACTCTTCGGAGTTCATTCA
***COX II***	17709	F- CCTGGTGAACTACGACTGCT
		R- GCTTGATTTAGTCGGCCTGG
***B***_***2***_***M***	12010	F-GACTCGCATAGGAACCTCATT
		R-CACATCAAAGCCCGCAGT
***U6***	19862	F-CTCGCTTCGGCAGCACA
		R-AACGCTTCACGAATTTGCGT
**miR-27b**	NA	F-TTCACAGTGGCTAAGTTCTGC
		R-primer (Uni-miR qPCR Primer, included in kit)
***MyHCⅠ***	140781	F-GCCTGGGCTTACCTCTCTATCAC
		R-CTTCTCAGACTTCCGCAGGAA
***MyHCⅡa***	17882	F-CAGCTGCACCTTCTCGTTTG
		R-CCCGAAAACGGCCATCT
***MyHCⅡx***	17879	F-GGACCCACGGTCGAAGTTG
		R-CCCGAAAACGGCCATCT
***MyHCⅡb***	17884	F-CAATCAGGAACCTTCGGAACAC
		R-GTCCTGGCCTCTGAGAGCAT
Foxj3T*	230700	F-CCGCTCGAGCGGAGTGCAGAGGGCCGTTGT
		R-AAATATGCGGCCGCTATAAACACCTGGTAAGGAGTGATGAT
***β-actin***	11461	F-TGGAATCCTGTGGCATC CATGAAAC
		R-TAAAACGCAGCTCAG TAACAGTCCG

Abbreviations for primers used throughout the study: *Foxj3*, forkhead box J3; *MEF2c*, myocyte enhancer factor 2C; *PGC1-α*, peroxisome proliferator-activated receptor gamma, coactivator 1 alpha; *NRF1*, nuclear respiratory factor 1; *mtTFA*, mitochondrial transcription factor A,; *ND2*, NADH dehydrogenase subunit 2; *ATP6*, ATP synthase F0 subunit 6; *COX I*, cytochrome c oxidase I; *COX II*, cytochrome c oxidase II; *B2M*, beta-2 microglobulin; *MyHCⅠ*, myosin heavy chain I; *MyHC IIa*, myosin heavy chain IIa; *MyHC IIb*, myosin heavy chain IIb; *MyHC IIx*, myosin heavy chain IIx; *Foxj3T* * describes the primer used for amplification of the binding site of the 3’UTR of *Foxj3*; U6 and β-actin were used as an endogenous control. NA, no Gene ID of Mus musculus miRNA-27b (mmu-miR-27b) in NCBI.

### Immunofluorescence analysis

Mitochondrial biogenesis was measured by using MitoTracker Green FM (Invitrogen, Carlsbad, CA, USA). Initially, a stock solution of MitoTracker was produced by dissolving the lyophilized MitoTracker in anhydrous dimethylsulfoxide to a final concentration of 1 mM. The stock solution was further diluted to a working concentration of 100 nM with GM. After C2C12 cells were transfected with the miRNA-27b mimic or inhibitor, cells were incubated for 30 min at 37°C with the Mitotracker Green working solution. Then the cells were washed twice with phosphate-buffered saline (PBS). Myogenic differentiation of C2C12 cells was determined by immunofluorescence staining of the slow skeletal muscle fiber myosin heavy chain. Briefly, C2C12 cells were cultivated in 12-well plates, where differentiation was induced. After 5 days, the cells were fixed with 4% paraformaldehyde for 20 min and treated with 0.2% Triton X-100 in PBS for 10 min at room temperature, then blocked with 5% normal goat serum in PBS for 1 h. Following incubation with monoclonal primary antibodies against mouse slow skeletal myosin heavy chain (Abcam, Shanghai, China) over night at 4°C, cells were incubated with a FITC- conjugated secondary antibody (Boster, Wuhan, China). Cells were then examined under a fluorescence microscope (Olympus, Kyoto, Japan). Quantification of green fluorescence was performed by LSM imaging software.

### Luciferase reporter assay

The wild-type 3’UTR of Foxj3 was amplified from genomic DNA of C2C12 cell (primers shown in Table 1), mutant-type Foxj3 3’-UTR was manufactured using commercial kits (TransGen Biotech, Beijin, China) according to the manufacturer’s instructions. The wild and mutant Foxj3 3’-UTR were inserted into the psiCHECK™-2 vector (Promega, Madison, WI, USA) between the *XhoI* and *NotI* restriction sites. Plasmids were sequenced afterwards (BGI, Shenzen, China) to verify correct insertion. For the luciferase reporter analysis, HeLa cells were cotransfected with empty psiCHECK™-2 plasmids or with the psiCHECK™-2 vector containing wild-type and mutant Foxj3 3’-UTR in conjunction with either mouse miRNA-27b mimic or a mimic control. After 24 h of transfection, luciferase activities were measured with the Dual-Glo Luciferase Assay System (Promega) following the manufacturer’s instructions.

### Western blotting

Proteins were extracted from C2C12 cells using lysis buffer (Sigma, Louis, Mo, USA) according to the manufacturer’s instructions. The wells of a 10% SDS-polyacrylamide gel were loaded with equal amounts of protein (20 μg), samples were then electrophoretically separated and finally transferred to a PVDF membrane (Bio-Rad, CA, USA). The membranes were hybridized with a primary antibody against Foxj3 (Santa Cruz, Santa Cruz, CA, USA), mitochondrial cytochrome c oxidase subunit II (COX II), voltage dependent anion channel (VDAC) and β-Actin (Boster, Wuhan, China), and incubated overnight at 4°C. Membranes were washed and treated with horseradish peroxidase-conjugated secondary antibodies (Boster), enzyme activity was then visualized with DAB substrate solution (Boster).

### Statistical analysis

Data were analyzed with SPSS (21.0 version). All data are presented as means ± standard deviation (S.D.). Differences between groups were analyzed with one-way ANOVA (three or more groups) or Student’s t-test (two groups). *P* < 0.05 was considered to be statistically significant.

## Results and Discussion

### Mitochondria content and miRNA-27b expression during C2C12 cell differentiation

**Fig 1:**
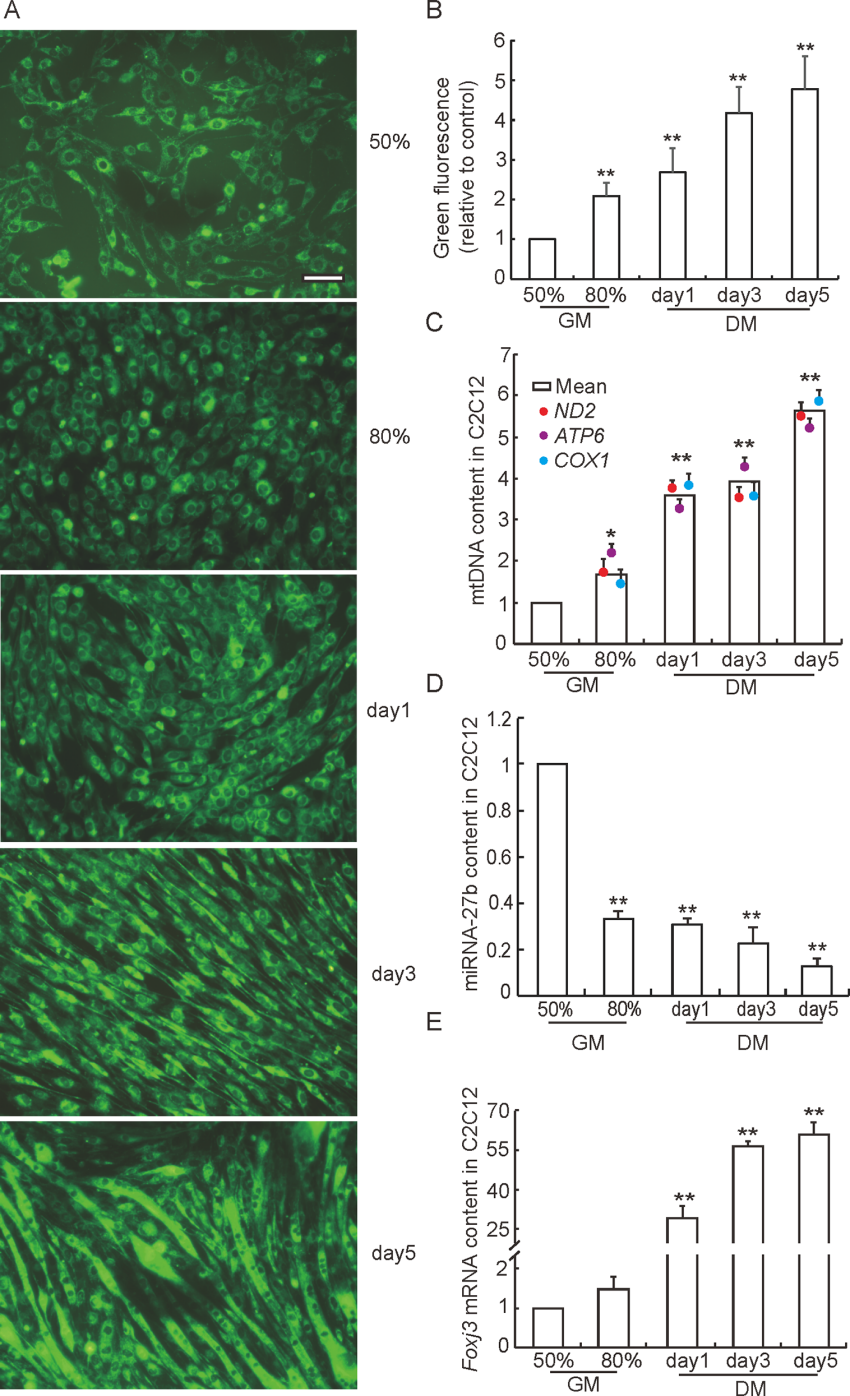
Mitochondria content and miRNA-27b expression during C2C12 cell differentiation. **(A)** The change of mitochondrial content during myocyte differentiation was visualized by fluorescence staining (MitoTracker Green FM). 50% and 80% refer to the degree of cell confluence. Scale bar: 20 μm. (B) Quantification of green fluorescence performed with LSM imaging software. Fluorescence intensities are normalized to those measured at 50% cell confluence. Data represent means ± SD of three independent experiments and each one performed in duplicate (n = 6). **(C)** Relative mitochondrial DNA copy numbers were measured in a RT-PCR assay and calculated as the ratio of mitochondrial gene expression (ATP6, COX1 and ND2) to the expression of the nuclear DNA single copy gene (B2M). All data represent mean of three independent experiments performed in duplicate (n = 6). **(D)** Relative value of expression levels of miRNA-27b in C2C12 cells during myogenic differentiation (RT-PCR) (n = 6). **(E)** Relative value of expression levels of *Foxj3* mRNA in C2C12 cells during myogenic differentiation (RT-PCR) (n = 6).GM: growth medium; DM: differentiation medium. Data are means ± SD; * *P* < 0.05, ** *P* < 0.01.

To explore the change of mitochondria content during C2C12 differentiation, mitochondria were stained with fluorescence tracker during thire differentiation from myoblasts to myotubes. As shown in Fig 1A and 1B, mitochondria content significantly increased during the process of differentiation (*P <* 0.01). In agreement with this observation, the mtDNA copy number increased approximately 6 times (Fig 1C), which is consistent with previous studies that pointed out that cells undergoing differentiation have higher energy demands than those in proliferation [10]. The expression of miRNA-27b was strongly decreased during the differentiation process (Fig 1D), which suggests that miRNA-27b may negatively affect mitochondrial biogenesis, and, indeed, previous reports support this hypothesis [25]. Those studies reported ectopic expression of miRNA-27a or miRNA-27b to impair mitochondrial biogenesis in adipose cells by target gene suppression. However, additional research is needed to prove the role of miRNA-27b in the regulation of the mitochondrial biogenesis in myocytes and muscle tissue. After performing a bioinformatic prediction, we identified *Foxj3* as a possible target gene of miRNA-27b, a fact that we verified in subsequent experiments. We found the expression of *Foxj3* to be sharply increased during C2C12 cell differentiation (Fig 1E). *Foxj3* is a forkhead/winged helix transcription factor, that regulates downstream gene expression through DNA-binding dependent mechanisms or, alternatively, through protein-protein interactions [26]. Moreover, *Foxj3* was found to be an upstream transcriptional activator of *Mef2c*, which, in turn, is a known regulator of the mitochondrial biogenesis [9,10,27]. These results indicate that miRNA-27b may negatively regulate the mitochondrial biogenesis in C2C12 cells by targeting the *Foxj3* gene.

### miRNA-27b directly targets to Foxj3

**Fig 2:**
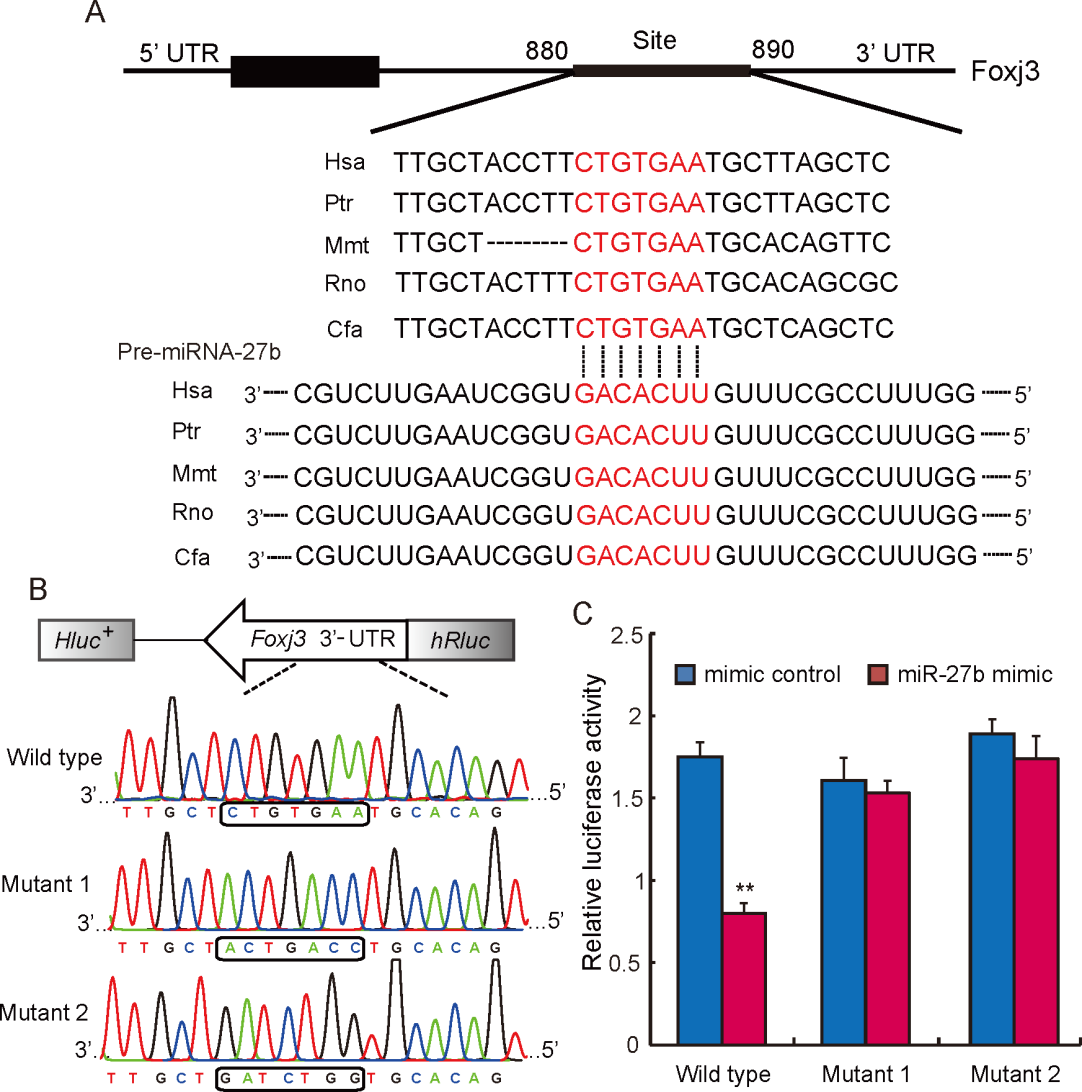
miRNA-27b targets the 3’-UTR of Foxj3. **(A)** Sequence alignment of miRNA-27b with 3’-UTR of human (Hsa), chimpanzee (Ptr), mouse (Mmu), rat (Rno), dog (Cfa) *Foxj3* mRNA. Binding site and seed region of miRNA-27b are indicated in red. **(B)** Recombinant luciferase reporter plasmid containing the wild-type 3’-UTR of *Foxj3*, or one of the two mutant 3’-UTR of *Foxj3*, respectively. Fragment insertion was verified by Sanger Sequencing (bottom). **(C)** The repressive effect of miRNA-27b on the activity of *Foxj3* as revealed in luciferase assays conducted with HeLa cells co-transfected with the wild-type or mutant Foxj3-3’UTR luciferase construct, and miR-27b mimic or mimic control. Data are means ± SD (n = 6), ** *P* < 0.01.

In order to investigate the target gene of miRNA-27b, we conducted a sequence alignment analysis. Our results show that the 3’-UTR of *Foxj3* mRNA contains a conserved complimentary binding site for the seed sequence of miRNA-27b, and the complimentary sequences were found to be highly conserved among different species (Fig 2A). Since these results supported our hypothesis that miRNA-27b may suppress *Foxj3* activity by binding to its 3’-UTR, we decided to confirm this assumption in luciferase reporter assays. One wild-type and two mutant *Foxj3* luciferase reporter plasmids were constructed, by inserting the 3’-UTR of *Foxj3* (Fig 2B). Co-transfection of the wild-type luciferase plasmid and miRNA-27b mimics in HeLa cells caused a significantly reduced luciferase activity when compared with the control group (*P* < 0.01). However, when co-transfection was carried out with either one of the two mutant plasmids, the relative luciferase activity was not significantly affected (Fig 2C). These results confirmed that the 3’-UTR of *Foxj3* is the direct target of miRNA-27b.

### The role of miR-27b in mitochondrial biogenesis

**Fig 3:**
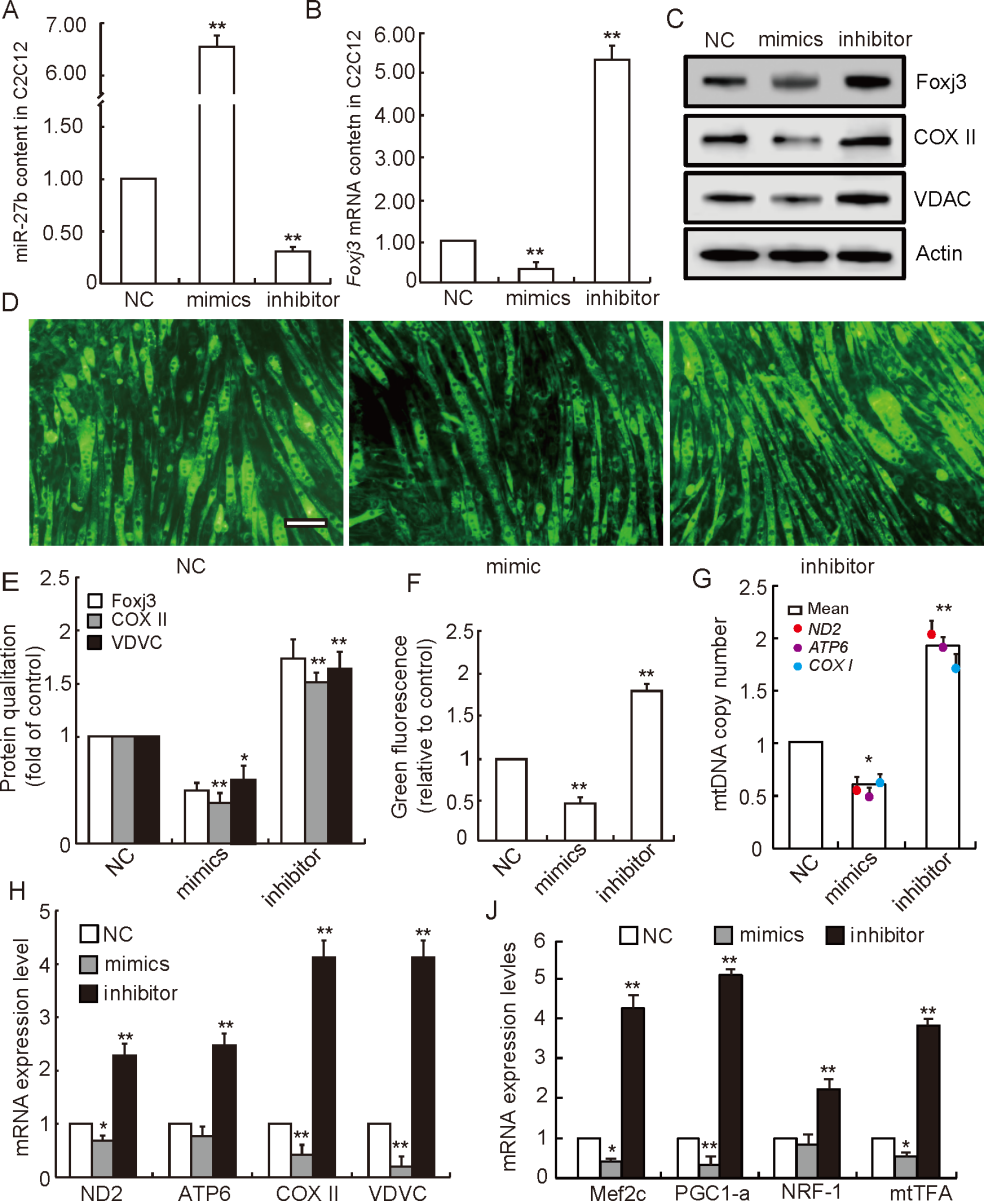
The role of miRNA-27b in mitochondrial biogenesis. Expression of miRNA-27b **(A)** and its target gene *Foxj3* **(B)** in C2C12 cells transfected with miRNA-27b mimic or inhibitor. Results are normalized to negative control (NC). Data represent means ± SD of three independent experiments performed in duplicate (n = 6). **(C)** Levels of proteins related to mitochondrial biogenesis as detected in Western Blots (n = 6). **(D)** Mitochondrial content of myoblasts with upon increase or reduction of miRNA-27b levels, visualized after fluorescence staining (n = 6). **(E)** Protein bands were quantified by densitometry and normalized to β-Actin levels (n = 6). **(F)** Fluorescence intensities normalized to negative controls as measured with the LSM imaging software.(n = 6). **(G)** Mitochondrial DNA copy numbers normalized to negative controls as detected in a RT-PCR approach (n = 6). **(H)** Relative expression levels of mitochondrial genes ND2, ATP6 and COX II and nuclear encoded mitochondrial-resident gene VDAC were detected by RTPCR (n = 6). **(J)** Relative expression levels of downstream genes of Foxj3 (Mef2c, PGC1-α, NRF-1 and mtTFA) in myoblasts displaying an augmented or reduced content of miRNA-27b (n = 6). NC (negative control, no sequence similarities to any reported mouse gene sequence), Scale bar: 20 μm, All data are means ± SD, *P < 0.05, **P < 0.01.

Transfection of miRNA-27b mimic or the respective inhibitor enabled us to up- or down-regulate miRNA-27b levels in C2C12 myoblasts and to further investigate its effect on mitochondrial biogenesis. Five days after transfection, the expression level of miRNA-27b in C2C12 cells transfected with miRNA-27b mimic was elevated about 7-fold when compared to negative controls (*P* < 0.01). The expression level of miRNA-27b in the inhibitor group, however, was significantly decreased (*P* < 0.01) (Fig 3A). At the same time, expression levels of *Foxj3* mRNA and protein behaved similarly and were found to diminish upon miRNA-27b overexpression and vice versa (Fig 3B and 3C and Fig 3E). These results confirm *Foxj3* to be the direct target gene of miRNA-27b and suggest that miRNA-27b may inhibit *Foxj3* expression both at mRNA and protein levels. Furthermore, we found the mitochondria content to be decreased in the miRNA-27b mimic group and increased in the inhibitor group when compared to the control group (Fig 3D and Fig 3F), which resembles the results regarding the mitochondrial DNA copy number (Fig 3G). To further verify whether miRNA-27b may inhibit mitochondrial biogenesis, we quantified the protein expression of a nuclear encoded mitochondrial-resident gene (VDAC) and mitochondrial-encoded gene (COX II), which are important components of mitochondria, necessary to maintain its function. We selected VDAC and COX II due to the close relation between the cell’s overall content of these two proteins and the mitochondrial mass [28]. We found the protein levels of VDAC and COX II to be significantly decreased in the miRNA-27b mimic group, whereas a significant increase was observed in the inhibitor group (Fig 3C and Fig 3E). These results are in accordance with those regarding the mRNA transcript of these two genes (Fig 3H). With regards to other two mitochondrial genes ND2 and ATP6, a significant reduction of mRNA levels could be observed for the former while only a trend to this effect was detectable for the latter when miRNA-27b overexpression (Fig 3H). We therefore conclude that miRNA-27b may not only inhibit mitochondrial biogenesis but may also further suppress the transcription activity of mitochondria in C2C12 cells. Even though a recent study has demonstrated that miRNA-27b may promote mitochondrial elongation by targeting the mitochondrial fission factor (Mff) in liver cells [29], we did not find miRNA-27b’s ectopic expression has the function of changing Mff expression in muscle cells. This functional difference may be related to the use of distinct cell lines. For example, Karbiener *et al*. found miRNA-27b could impair human adipocyte differentiation by targeting *PPARγ* [30]. However, Wang *et al*. proved miRNA-27b able to induce cardiac hypertrophy and dysfunction in mice [31].

**Fig 4:**
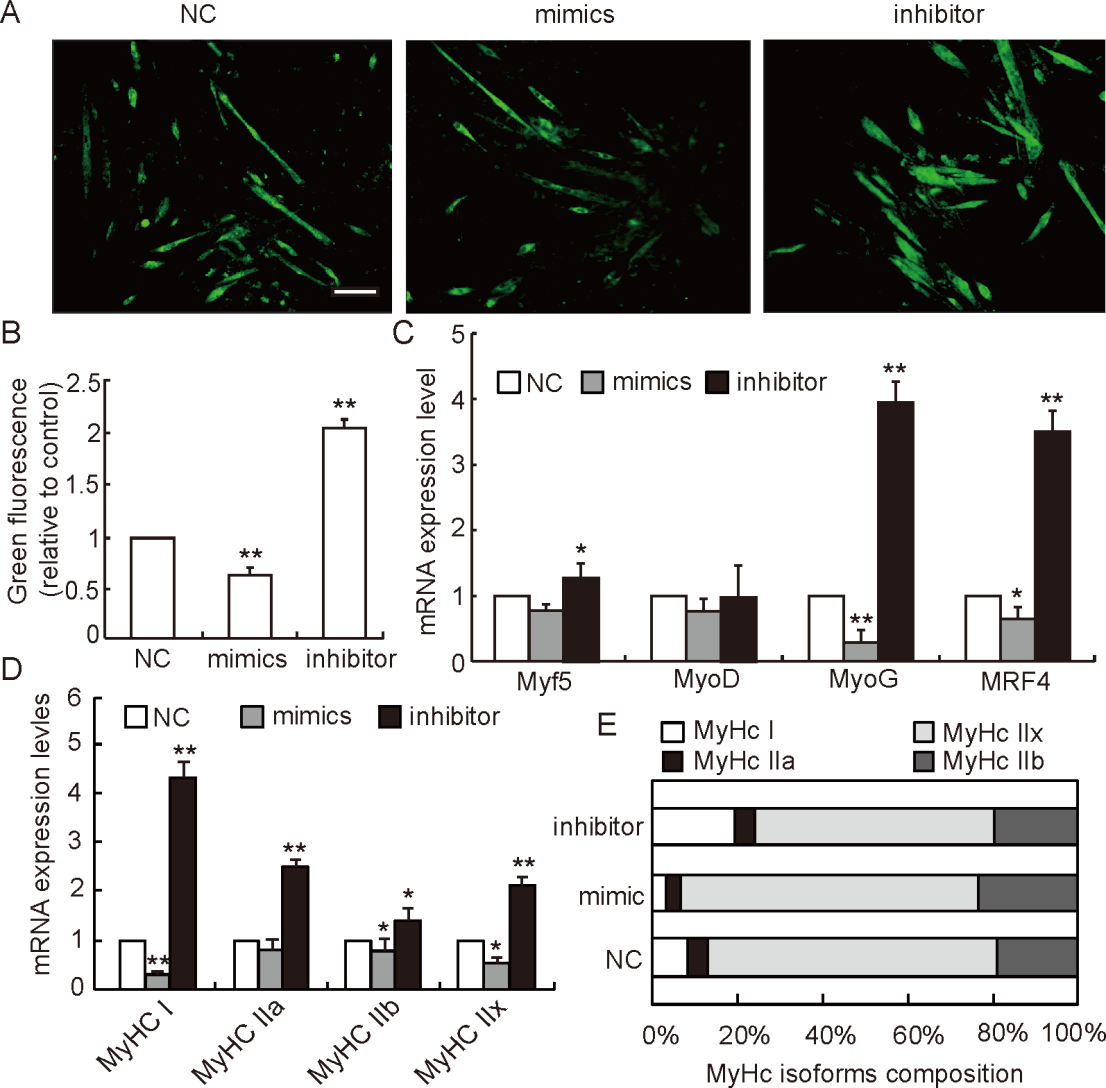
miRNA-27b influences myogenic differentiation of C2C12 myoblasts. **(A)** Effects of miRNA-27b on slow muscle fiber type formation in C2C12 cells as evaluated after immunofluorescence staining of MyHC I 5 days after differentiation. Scale bar: 20 μm. **(B)** Green fluorescence intensities normalized to negative controls as measured with the LSM imaging software. Data represent means ± SD of three independent experiments performed in duplicate (n = 6). **(C)** Relative expression levels of myogenic regulatory factors *Myf5*, *MyoD*, *MRF4* and *MyoG* in cells with an augmented or reduced content of miRNA-27b as detected by RT-PCR (n = 6). **(D)** Relative expression levels of myosin heavy chain isoforms *MyHC I*, *MyHC IIa*, *MyHC IIb* and *MyHC IIx* upon increase or reduction of miRNA-27b expression as detected by RT-PCR (n = 6). **(E)** Composition of myosin heavy chains (*MyHC I*, *MyHC IIa*, *MyHC IIb* and *MyHC IIx*) in cells with an augmented or reduced expression of miRNA-27b as detected by RT-PCR (n = 6). NC (negative control, no sequence similarities to any reported mouse gene sequence), All data are means ± SD, *P < 0.05, **P < 0.01.

**Fig 5:**
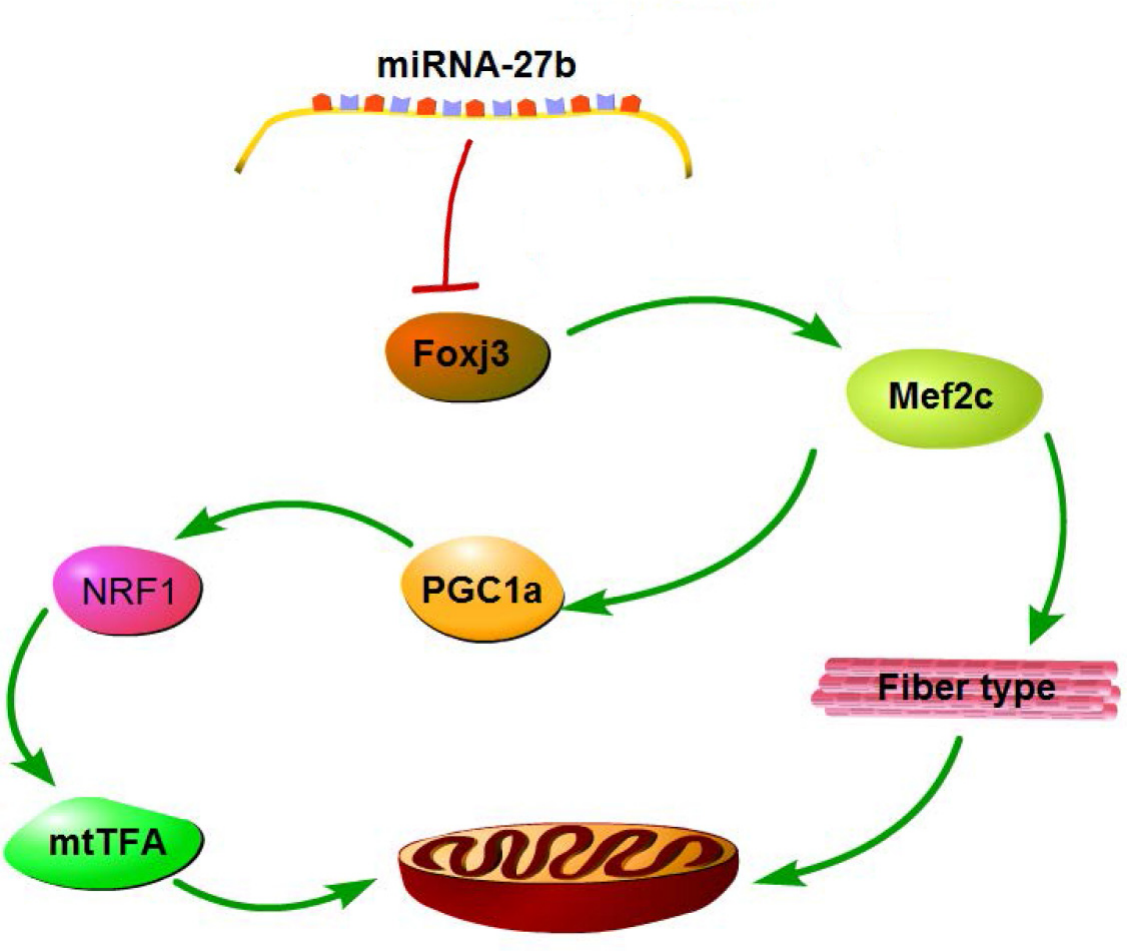
Possible pathway used by miRNA-27b to suppress mitochondria biogenesis. *Foxj3* and *Mef2c* are target genes of miRNA-27b and are suppressed by it. The expression of downstream gene of *Foxj3* (*Mef2c*, *PGC1c*, *NRF1* and *mtTFA*), which play important roles in promoting mitochondrial biogenesis, is reduced upon suppression of *Foxj3* by miRNA-27b. *Mef2c* and *PGC1* also have other functions that may alter the slow fiber type composition and thus diminish their mitochondria content.

In our study, expression levels of downstream genes of *Foxj3* (*Mef2c*, *PGC1α*, *NRF1* and *mtTFA*) were evaluated to shed more light on the pathway possibly used by miRNA-27b to suppress mitochondrial biogenesis. As shown in Fig 3J, expression levels of all those genes were significantly decreased in the myotubes of the miRNA-27b overexpression group, while down-regulation of miRNA-27b had the opposite effect. Matthew *et al*. found *Mef2c* to be a direct downstream target gene of *Foxj3* in myoblasts [27]. *Mef2c* may bind to the promoter of *PGC1α* and mediate the transcriptional activation of *PGC1α* to stimulate the biogenesis of mitochondria [9]. *PGC1α* has a strong positive effect on the expression of *NRF1* and *NRF2* genes, and forms a coactivated transcription factor after binding to *NRF1*. Then the complex binds to the promoter of mtTFA and directly regulates the process of mitochondrial DNA replication and transcription [32]. Moreover, various studies speculated that *Mef2c* and *PGC1α* may promote slow type fiber formation [33,34], which are well known for their higher mitochondrial content and density [35]. Therefore, the suppression of mitochondrial biogenesis by miRNA-27b potentially influences myogenic differentiation and muscle fiber type composition by decreasing *Mef2c* and *PGC1α* expression. We found that overexpression of miRNA-27b could decrease slow muscle fiber synthesis (Fig 4A and 4B), which is consistent with the expression of myogenic regulatory factors MyoG and MRF4, which are mainly involved in the control of myogenic differentiation at intermediate and later stage and that are required for the myotube formation [36]. Interestingly, we also found that overexpression of miRNA-27b could diminish the synthesis of all types of myosin heavy chains, but was most efficient reducing the synthesis of MyHC I (Fig 4D), which, in turn, resulted in a reduction of the percentage of slow muscle-fiber (Fig 4E). Therefore, we deduce that miRNA-27b may suppress the mitochondrial content of myotubes by decreasing thire slow type fiber content. In addition, Chinchilla *et al*. reported that miRNA-27b also targeted *Mef2c* and played an important role in heart development [37]. This implies that miRNA-27b may simultaneously target *Foxj3* and *Mef2c* to regulate mitochondria biogenesis. These data elucidate a possible pathway used by miRNA-27b to regulate mitochondria biogenesis and function. A plausible scheme is shown in Fig 5.

## Conclusion

Our results point out an important role of miRNA-27b in mitochondria biogenesis in myocytes. We have shown miRNA-27b to be able to suppress the mitochondria content in myoblasts and to subsequently reduce the expression of some mitochondrial genes by targeting *Foxj3*. Overexpression of miRNA-27b decreased the expression of Foxj3 downstream genes *Mef2c*, *PGC1α*, *NRF1* and *mtTFA*, which have a vital function in accelerating the biogenesis of mitochondria. Our findings reveal a possible mechanism of how miRNAs regulate mitochondria biogenesis in myocytes.
